# Effects of Loving-Kindness Meditation on Doctors’ Mindfulness, Empathy, and Communication Skills

**DOI:** 10.3390/ijerph18084033

**Published:** 2021-04-12

**Authors:** Hao Chen, Chao Liu, Xinyi Cao, Bo Hong, Ding-Hau Huang, Chia-Yi Liu, Wen-Ko Chiou

**Affiliations:** 1 Graduate Institute of Business and Management, Chang Gung University, Taoyuan 33302, Taiwan; haochen19606@163.com (H.C.); victory666666@126.com (C.L.); 2 College of Aviation, Huaqiao University, Xiamen 361021, China; 3 Clinical Neurocognitive Research Center, Shanghai Key Laboratory of Psychotic Disorders, Shanghai Mental Health Center, Shanghai Jiao Tong University School of Medicine, Shanghai 200030, China; rekixinyicao@163.com; 4 Department of Psychiatry, Shanghai Mental Health Center, Shanghai Jiao Tong University School of Medicine, Shanghai 200030, China; crystalhongbo@163.com; 5 Institute of Creative Design and Management, National Taipei University of Business, Taoyuan 22058, Taiwan; hau1012@gmail.com; 6 Department of Psychiatry, Chang Gung Memorial Hospital, Taipei 10507, Taiwan; liucy752@cgmh.org.tw; 7 Department of Industrial Design, Chang Gung University, Taoyuan 33302, Taiwan

**Keywords:** loving-kindness meditation, mindfulness, empathy, communication skills

## Abstract

Background: In the context of increasing doctor–patient tensions in China, the objective of this study was to explore and examine the effects of loving-kindness meditation (LKM) on doctors’ mindfulness, empathy, and communication skills. Methods: A total of 106 doctors were recruited from a hospital in China, and randomly divided into an LKM training group (n = 53) and waiting control group (n = 53). The LKM training group received 8 weeks of LKM training intervention, whereas the control group received no intervention. Three major variables (mindfulness, empathy, and communication skills) were measured before (pre-test) and after (post-test) the LKM training intervention. Results: The empathy and communication skills of the LKM group were significantly improved compared with those of the control group, but the level of mindfulness did not significantly change. Conclusions: The results suggested that LKM may contribute to improving physicians’ empathy and communication skills. However, the mechanisms that underlie the effects of the LKM on mindfulness, empathy, and communication skills and other psychological constructs needs further elucidation.

## 1. Introduction

As a result of societal development, the progress of medical technology, and the gradual improvement of people’s living standards, people’s awareness of healthcare has been constantly enhanced, and their demands for medical services have gradually increased. Due to the increasing medical and health needs of the people, providing high-quality medical services and building a harmonious doctor–patient relationship has become one of the main goals of the current development of public health [1]. Doctor–patient communication is of great significance in medical services. Effective communication between doctors and patients is the premise to ensure medical quality and patient satisfaction. In recent years, poor communication and strained relations between doctors and patients have been common in China. Due to the lack of effective communication, medical disputes and conflicts are common. Doctor–patient disputes are increasing, the doctor–patient relationship has deteriorated sharply, and incidents, such as violence against doctors, occur frequently, which has aroused wide concern from all sectors of society [2,3].

It was found that, from 2003 to 2013, there were 101 cases of serious violence against doctors, among which 24 doctors died. The frequency of these incidents has increased. These incidents of violent injury and injury were most serious in emergency departments and most frequent in tertiary hospitals, with a high incidence of 66% in China [4]. The Chinese Medical Doctors Association released the White Paper on the Practice of Doctors in China. According to the survey, nearly 60% of medical workers have experienced verbal violence, 13.07% have suffered physical injury, and only 27.14% have not experienced violence. Each hospital has an average of 27 violent injuries per year. The incidence of physical attacks and obvious injuries on doctors has increased annually, and the vast majority of doctors has been subjected to psychological violence, such as abuse and threats [5]. At present, more than 10,000 doctors are beaten and injured each year in China, and 73.33% of hospitals have reported the beating and abuse of doctors by patients and their families. A share of 78.01% of doctors do not want their children to study medicine or become doctors. In 2019, Chinese courts concluded 155 cases of violent killing or injuring of doctors [1]. The Lancet has urged for an end to violence against Chinese doctors, calling on the government to investigate the root causes of physician–patient violence and propose effective measures to end the threat to doctors’ personal safety [6]. Under the circumstances of limited medical resources, heavy workload, and unsatisfactory salary, it is difficult for Chinese doctors to consistently ensure the quality of their work and service, and poor doctor–patient relationships are almost inevitable [7]. Violence in medical places, ranging from verbal abuse to the murder of doctors, not only reduces patients’ trust in doctors, but also represents a failure to guarantee doctors’ safety and personal dignity. This is a significant threat to the positive development of medical and health services, and undermines the current medical environment and social stability in China [8,9].

### 1.1. Patient-Centered Care

Traditional medical diagnosis and treatment tend to regard patients as biological people in epistemology, but ignore patients’ social attributes. In practical work, the focus is often only the diseased organs, and the overall system of the human being is ignored. Physicians attach importance to physical factors over psychological and social factors. In medical scientific research, more attention is paid to the biological process of the body, with less focus on the psychological process of patients, and the important role of psychosocial factors on health is ignored [10]. Classical medical science regarded the human body as a machine; disease was seen as a fault of the machine, and the job of the physician was to repair this machine. Medical personnel may also focus on the disease and ignore the “patients” and the patient’s feelings, and are thus unable to use the biopsychosocial medical model to diagnose and treat diseases [11]. Physicians may only pay attention to the improvement of their own medical level, ignoring the communication with patients. Data on patients’ biochemical indexes is obtained using various medical instruments, which has increasingly become an important basis for disease diagnosis and treatment. This tendency to rely more on modern scientific instruments has gradually weakened the exchange of ideas between doctors and patients, ignoring the influence of social, psychological, and other factors on patients’ diseases. This lack of humanistic care in medical activities has exacerbated the deterioration of the doctor–patient relationship [12]. The current medical education system focuses mainly on medical skill education, and humanistic education is insufficient, which can lead to a low sense of responsibility, lack of patience and compassion, and poor service attitude among medical staff. In medical services, medical personnel who master the medical professional knowledge and skills, and patients who have little medical knowledge, form a special and unequal interpersonal relationship [13]. Due to the imbalance of medical information between doctors and patients, medical personnel are in a dominant position regarding medical information and disease diagnosis and treatment, whereas patients are in a relatively inferior position. In the process of doctor–patient communication, medical personnel are often shown to be the authority and leader because of their professional knowledge and technology, their right to diagnose and treat diseases, and their right to control medical and health resources. As a result, medical staff may communicate with patients and their families in a serious and commanding tone, reflected in a paternalistic style of doctors, and causing dissatisfaction among patients [14]. Due to the increasingly fierce competition in the provision of medical services, patients may not only pay attention to the medical level of hospitals, but also to their humane services. Patients hope to receive transparent, personalized, and dignified medical care, in which they are recognized and respected. In addition to elements of “hard power”, such as technology and equipment, hospitals should also address elements of “soft power”, such as the management mode and service level [15]. The integration of humanization into the overall process of medical service and hospital management, changing outdated ideas that hospitals only treat diseases, and establishing the modern medical idea that the medical object is firstly “patients”, and then “the disease”, are important considerations in modern hospital construction. Hospitals should gradually transform the “disease-centered” mode into a “patient-centered care” mode, enhance service awareness, provide more humanistic care to patients, and continuously improve patient satisfaction through humanized services [15].

The concept of patient-centered care includes paying more attention to the patient’s perspective and promoting the patient’s participation in their care to provide the patient with greater control over their health. Many different definitions of the concept of patient-centered care have been proposed, but all of them include the main drivers of patient-centered communication and are characterized by the empathy of the physician [16]. Good doctor–patient communication, empathy, and a non-judgmental attitude are key factors in patient-centered care [17].

Strengthening doctor–patient communication is the most effective way to enhance and maintain a good doctor–patient relationship [10]. In doctor–patient communication, both doctors and patients focus on the injury, diagnosis and treatment, health, and related factors in medical and healthcare services. This is dominated by health providers who, based on various characteristics of the comprehensive information of multi-channel communication, scientifically guide diagnosis and treatment in patients. The aim is that doctors and patients reach a consensus to build trust and cooperative relations, to maintain human health and promote the aim of medical development and social progress [11]. The interpersonal relationship model emphasizes that good doctor–patient communication is not only beneficial for doctors to establish good doctor–patient cooperation and conduct normal medical procedures, but it can also reduce patients’ pain and anxiety. Conversely, poor communication may cause doctors to experience psychological pressure, dissatisfaction, and fear of patients, increase medical risk, and reduce patient satisfaction [12].

Empathy refers to the ability to understand and experience the inner world of patients, which is commonly referred to as transpositional consideration [18]. Specifically, empathy in doctor–patient communication refers to: (1) With the help of the patient’s words, expressions and behaviors, physicians strive to penetrate into the patient’s inner world, judge another person’s feelings by one’s own, and share the patient’s emotional experience. (2) Understanding the connection between the patient’s various psychological activities, and the connection between the patient’s emotions and their experience and personality. (3) Physicians convey their understanding of the patient to obtain the patient’s approval. Patients feel a need to be understood, and not being understood can be painful for them. Understanding can make an immeasurable difference to a person’s physical and mental health. Doctors should always be empathetic in medical situations and aim to understand patients’ feelings [19]. Therefore, doctors must strengthen their self-training and improve their abilities of mindfulness, empathy, and doctor–patient communication.

Non-judgment is an important characteristic of mindfulness. Mindfulness is an open, conscious experience of the present moment. It emphasizes an uncritical treatment of ongoing matters and experiences. Studies have shown that mindfulness is a potential predictor of doctor–patient communication, which can be effectively predicted by the self-monitoring dimension of mindfulness [20]. Doctors who practice mindfulness or attend mindfulness communication classes listen more actively to their patients, gain a deeper understanding of their experiences, and think more from their patients’ perspectives. Patients also believe that doctors are more patient-centered in communication. It can be seen that practicing mindfulness and improving the communication level of mindfulness at work may be an effective means to establish and maintain a good doctor–patient relationship [21]. Therefore, physicians must strengthen self-training, doctor–patient communication skills, and empathy and mindfulness, which are three key competencies in patient-centered care.

### 1.2. Loving-Kindness Meditation

Loving-kindness meditation (LKM) is a form of compassion-based meditation that has been practiced for more than 2500 years. However, research has only recently begun to explore its role as a psychological intervention. The meaning of LKM is to care for oneself and all creatures. Its purpose is to cultivate unconditional feelings of love, kindness, and acceptance [22]. In the practice of LKM, the practitioner directs kindness first to himself, then to loved ones, acquaintances, strangers, and, finally, to all sentient beings. LKM has many effects and can be practiced at any time in a variety of positions, such as lying down, sitting, and walking. Compared with traditional meditation methods, LKM is a simpler method that is not limited by time and space, and can be performed at any time and in any place [23]. Therefore, LKM is suitable for doctors. When doctors encounter emergencies, they can also use LKM to calm their emotions and resolve conflicts in a timely manner.

As a mindfulness-based meditation method, the concept of LKM incorporates elements of mindfulness and empathy. However, few studies have examined the effects of LKM on doctor–patient communication, doctor empathy and doctor mindfulness. Sorensen’s research showed that LKM can improve the mindfulness of college students [24]. Mindfulness is purposefully, consciously paying attention to and perceiving everything in the present moment without any judgment, analysis, or reaction, but simply perceiving and noticing. Mindfulness training enables participants to face rather than escape potential difficulties. Participants develop an open, receptive attitude to dealing with current thoughts and emotions. This is all achieved via meditation, and the core technique is to focus, to be aware of your physical and emotional states, and to let nature take its course without judgment. This mindfulness practice promotes a pattern of consciousness awakening rather than a habituated, automated pattern of negative thinking [25]. Previous research has shown that mindfulness increases the ability to communicate between business leaders [26], nurses [27], and family members of cancer patients [28]. Although there is much evidence that mindfulness is positively associated with doctor–patient communication, research exploring the mechanisms underlying this relationship is limited. Empathy may help explain the relationship between mindfulness and doctor–patient communication, which is an issue that deserves further investigation. Previous studies have explored the preconditions of empathy and found that mindfulness can promote empathy in individuals [29]. Mindfulness has been found to improve empathy among counselors [30], adolescents with a history of childhood abuse [31], college students [32], adults [33], and couples [34]. Researchers have examined specific groups of people and found that mindfulness enhanced the empathy of nurses [35] and ICU staff [36]. Literature has also revealed the positive effects of empathy on communication. Studies have shown that empathy can enhance the communication ability of social workers [37], and empathy is also positively correlated with the communication abilities of maternal partners and Japanese college students.

Researchers have also examined employees and found that empathy was positively correlated with the communication abilities of midwives [38], oncology nurses [39], and British police officers [40]. Overall, these previous findings strongly suggest that people with high levels of mindfulness experience higher levels of empathy, and can increase doctor–patient communication. To summarize, LKM should be an effective intervention method and have a positive impact on doctor–patient communication, and the level of empathy and mindfulness of the physician community.

### 1.3. Research Gap, Purpose and Hypotheses

During the COVID-19 outbreak in early 2020, the treatment by doctors of patients of the disease led to a positive transformation of mutual understanding and high coordination between doctors and patients. The difficulties in the relationship between doctors and patients were significantly alleviated. However, ensuring that this good doctor–patient relationship becomes a normal condition, rather than a temporary phenomenon during the epidemic period, is a topic worthy of further study. The most effective means to enhance and maintain a good doctor–patient relationship is to enhance doctor–patient communication and establish a good communication mechanism. Listening, empathy, and being patient-centered are all effective means to build a better doctor–patient relationship. We believe that by improving doctors’ empathy, mindfulness, and communication skills through LKM, the relationship between doctors and patients will be maintained in a virtuous dynamic cycle in the post-pandemic era, thus promoting the healthy, positive, and harmonious development of the society. Therefore, the purpose of this study was to verify whether LKM can enhance doctors’ mindfulness, empathy, and communication skills.

Based on the above arguments and evidence, we propose the following hypotheses:

**Hypothesis** **1** **(H1).** *For mindfulness, empathy, and communication skills, the post-test scores were significantly higher than the corresponding pre-test scores in the experimental (LKM) group.*

**Hypothesis** **2** **(H2).** *For mindfulness, empathy, and communication skills, there was no significant difference between the post-test scores and the corresponding pre-test scores in the control group.*

**Hypothesis** **3** **(H3).** *For mindfulness, empathy, and communication skills, the post-test scores in the LKM group were significantly higher than those in the control group.*

## 2. Methods

### 2.1. Participants

The subjects of this study were doctors working in a hospital in China. We published recruitment information through the internal network of the hospital. Finally, 106 eligible subjects were recruited, with an average age of 38.57 years (SD = 7.41), and 49 (accounting for 46.2%) were male. The subjects were randomly divided into two groups: the LKM training group (53 participants) and the waiting control group (53 participants). There was no significant difference in demographic factors such as age composition and sex ratio composition between the two groups (Table 1).

### 2.2. Instruments

The Mindfulness Attention Awareness Scale (MAAS). This scale is used to measure the individual trait of mindfulness, and was developed by Brown and Ryan [41]. The Chinese version of MAAS was adapted by Deng et al. [42], which has the same single factor structure of 15 items and a six-point Likert scale, where 1 to 6 mean “almost always” to “almost never”. Higher scores represent greater mindfulness. Among the many instruments for measuring the trait of mindfulness, MAAS is the most widely used [43]. A large number of studies have shown that MAAS has good reliability and validity in people with different cultures [44]. In the current study, Cronbach’s alpha was 0.92.

The Jefferson Empathy Scale (JSE). This scale is used to measure doctors’ empathy. First developed by Mohammadreza Hojat at Jefferson Medical School in 2001 [45], the scale is used to measure the empathy of medical staff in clinical practice and includes three aspects: compassionate care, perspective taking, and standing in the patient’s shoes This scale includes 20 items, and 10 reverse items that use negative words. Each item is rated on a scale of 1 to 7: a score of 1 means “strongly disagree”, and a score of 7 means “strongly agree”. The higher the score, the more empathetic the ability. The Chinese version of the JSE is used to measure doctors’ sympathy [46]. Previous studies have shown that the JSE has good validity [47]. In this study, Cronbach’s alpha was 0.91.

Liverpool Communication Skills Assessment Scale (LCSAS). This scale is used to measure doctors’ communication skills, and was developed by Humphris and Kaney [48]. It consists of the 5 dimensions of introductions, nonverbal behavior, respect and empathy, questioning, and giving information, and includes 12 items. A four-point Likert scale was adopted, with 0–3 representing unacceptable, poor, acceptable, and good. The scale has been widely used in clinical practice. The Chinese version of the LCSAS has been used to measure doctors’ communication skills [49]. Previous studies have shown that the LCSAS has good validity [50]. In this study, Cronbach’s alpha was 0.93.

### 2.3. LKM Intervention

The LKM intervention in this study was conducted by group training in 90 min sessions. Each session involved three successive stages: (1) a maximum of 15 min of psychological education, covering topics such as an introduction to LKM; (2) LKM exercise for 30 min; (3) a final discussion stage that lasted for 45 min in which participants could share their LKM experience with other participants and lecturers. The LKM intervention required practice in a quiet environment. The general procedure is to look inward, by focusing on the breath, and to remove all external distractions. When attention is stabilized, participants imagine a person they identify with who makes them feel warm and secure (usually a loved one) by saying things like “May you live in joy, may you live in peace. I wish you to be free from all physical and mental suffering.” to allow themselves to continue to experience peace and happiness. During this experience, the participants put all of their spirit into the feeling, and maintain it for a period of time. As the practice deepens, the practitioner expands the sense of comfort and contentment from near to far. The specific procedure is, first, to themselves; second, to loved ones; third, to a neutral person (emotionally neutral to oneself, i.e., neither like nor hate); fourth, to people that disgust them; and, then, to the entire universe. Finally, participants were asked to practice these formal meditation sessions between classes and to use the kindness and love technique in their workplace [51].

### 2.4. Procedure and Design

**Figure 1:**
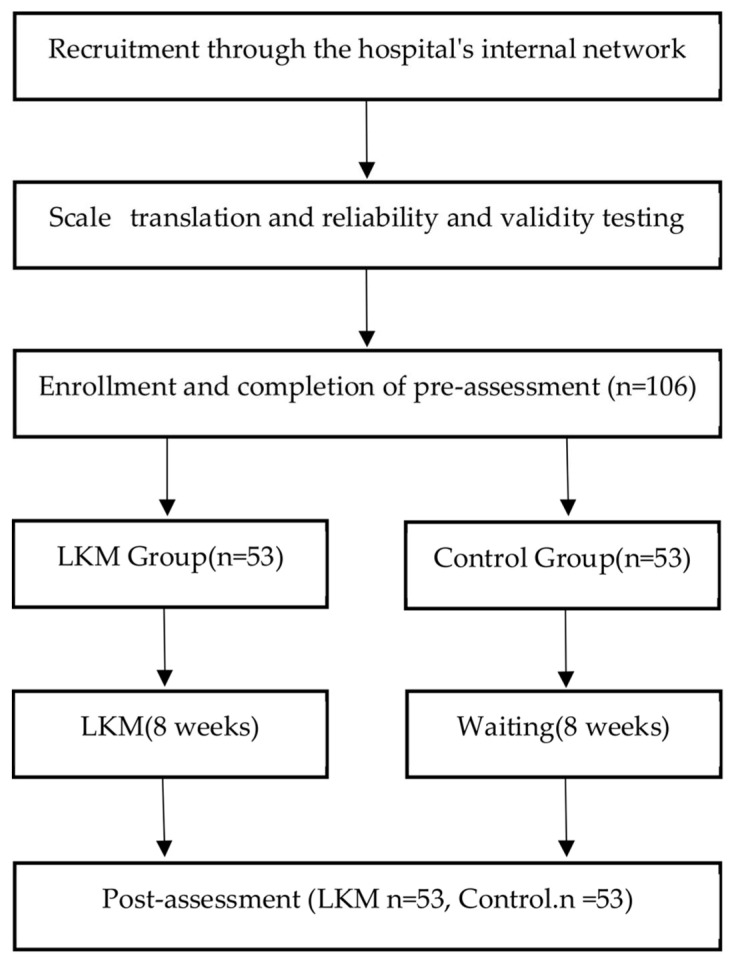
Procedure flow chart.

We posted a recruitment advertisement for LKM on the internal network of a hospital in China, stating that LKM is a self-exploration activity to help doctors better understand themselves and solve current problems. Physicians interested and eligible to participate in our LKM study provided their registration information. The inclusion criteria were: (1) practiced as a doctor for at least 3 years, and (2) could speak and read Chinese and were able to complete the questionnaire. Exclusion criteria were: (1) self-reported diagnosis of depression, anxiety, bipolar disorder, substance abuse, or attempted suicide by a medical professional; (2) previous experience of LKM. We then randomly assigned 106 eligible participants to either the experimental group (i.e., the LKM intervention group, 53 participants) or the control group (i.e., the waiting group, 53 participants). The study was conducted by researchers who had 10 years of practicing LKM and 2 years of teaching LKM. The meeting (LKM or waiting groups) was held in a quiet, plain room, with each participant given RMB 50 at the start of the survey to boost their motivation. At the beginning of the survey, the participants were provided with instructions to make sure they understood them before continuing. After confirming that they understood the instructions, the participants provided demographic information and completed the following questionnaire (pre-test): (1) Mindfulness Attention Awareness Scale (MAAS); (2) the Jefferson Empathy Scale (JSE); and (3) Liverpool Communication Skills Assessment Scale (LCSAS). The time required to answer the questionnaire was about 20 min. After all participants finished the pre-assessment, they were randomly allocated to the LKM group or the control group based on a computer-generated randomizer with matching for gender variables. The LKM group received LKM intervention 3 times per week for a total of 8 weeks, whereas the control group did not undergo any intervention. Many previous studies on LKM have used an intervention period of 8 weeks [52,53,54,55]. Thus, this study referred to the previous research to set the total length of the intervention. At the end of the intervention period (week 8), participants completed the same questionnaire again (post-assessment) and were given an additional RMB 50 as compensation (illustrated in Figure 1).

This study followed the funnel debriefing procedure. Participants were asked if they knew the purpose of the study and the topics that were being investigated, and if they were aware of the same questions in the pre-test and post-test. To enroll subjects who were blind to the experimental conditions and naively undertook the LKM exercises, funnel debriefing helped to obtain a homogeneous sample in both groups. Participants had the opportunity to record any number of questionnaires anonymously, and were able to exit at any stage. The study was approved by the Ethics Committee of Huaqiao University (IRB No.: 202002236B7) and the protocols were carefully reviewed to ensure that they complied with the ethics code of China Psychological Association (see Figure 1).

## 3. Results

**Figure 2:**
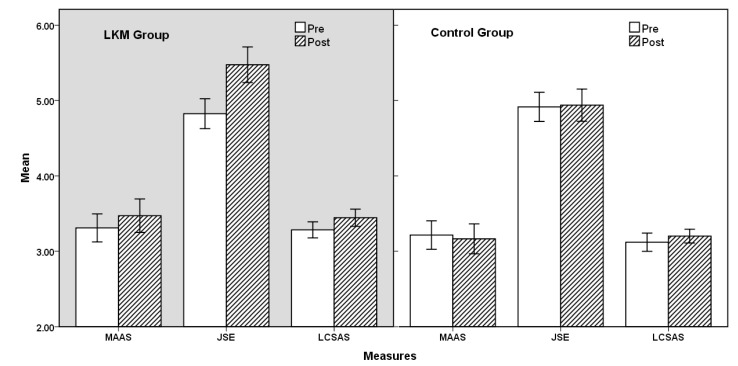
Comparison of three measures between the loving-kindness meditation (LKM) and control groups. MAAS, Mindfulness Attention Awareness Scale; JSE, the Jefferson Empathy Scale; LCSAS, Liverpool Communication Skills Assessment Scale.

We performed repeated-measured ANOVA on the three parameter estimates (mindfulness, empathy, and communication skills), with time (pre, post) as the within-subjects variable and group (LKM, control) as the between-subjects variable. Statistical details are reported in Table 2 and Figure 2.

For mindfulness, there were no significant main effects of time: F(1,104) = 0.427, *p* = 0.515, which showed that there was no significant difference in MAAS scores between the pre-test and the post-test. In addition, there were no significant main effects of group: F(1,104) = 3.251, *p* = 0.074, which revealed no significant difference between LKM and the control group. Time and group also did not show any significant interaction: F(1,104) = 1.544, *p* = 0.217. The results suggest that the LKM intervention did not significantly improve the participants’ mindfulness, which is not consistent with the hypotheses.

Empathy not only showed a significant main effect in time: F(1,104) = 9.290, *p* = 0.003, η^2^*_p_* = 0.082, it also showed a significant main effect of group: F(1,104) = 4.948, *p* = 0.028, η^2^*_p_* = 0.045. In addition, time and group also showed a significant interaction effect: F(1,104) = 8.089, *p* = 0.005, η^2^*_p_* = 0.072. This result shows that the LKM intervention significantly improved the empathy level of the subjects, which is consistent with the hypotheses.

Regarding communication skills, both time (F(1,104) = 6.272, *p* = 0.014, η^2^*_p_* = 0.057) and group (F(1,104) = 11.653, *p* = 0.001, η^2^*_p_* = 0.101) showed significant main effects, but the interaction effect between the two was not significant: F(1,104) = 0.686, *p* = 0.409. The results revealed that LKM intervention can significantly help to improve subjects’ communication skills, which is also consistent with the above hypotheses.

Our hypotheses stated that LKM should increase mindfulness, empathy, and communication skills. As the results showed that the LKM significantly increased participants’ empathy and communication skills, Hypothesis 2 was supported, and Hypotheses 1 and 3 were partially supported.

## 4. Discussion

### 4.1. LKM Can Improve Empathy and Communication Skills

There were significant differences between the empathy scores before and after the test. LKM enabled the physician to empathize with the patient, to correctly experience the emotional feelings of the patient, and to communicate with the patient with transpositional consideration. Thus, the patient knows that the physician can acknowledge the patient’s suffering without prejudice and evaluation [56]. The process involves the doctor constantly responding to the patient, to more deeply understand and produce therapeutic effects: first, the doctor perceives and understands the patient’s inner emotions and worries. Then, the doctor undergoes an internal experience, feels the pain and annoyance of the patient, further understands the kind of emotion and annoyance felt by the patient, and has a richer understanding of the patient’s emotions and thoughts. Finally, the kay factor is the physician’s actions in the process of empathy, and how they respond and guide the patient to induce a therapeutic effect. In the process of communication, doctors increasingly understand patients’ feelings and concerns. At this time, treatment often has multiple directions. Doctors guide the direction of treatment through response, such as guiding patients to accept their own diseases, to reduce their defense, and to master their diseases or symptoms. With a compassionate response, doctors can guide the patient towards the potential to achieve a positive treatment direction [57]. In terms of the mechanism by which LKM affects empathy, Preston and de Waal proposed a perception–action model of empathy(PAM). According to this model, an individual’s perception of the behavior of others will automatically activate the representation of his personal experience related to that behavior. Therefore, when an individual perceives the action or emotion of others, the portion of the brain that represents the corresponding action or emotion will be automatically activated, thus making the individual produce the representation of the same form [58]. Subsequently, scholars found that the mirrored nervous system can transform the observed activities and experiences of others into the form of the system’s own neural activities, leading to the generation of the same or similar movements and experienced feelings. This provides neurobiological evidence for the PAM, which is also believed to be the possible neural mechanism of empathy [59]. In addition, Lutz et al. used human sounds, such as positive noises (baby’s laughter), neutral noises (restaurant noise), and negative noises (woman’s pain), to stimulate 15 participants with experience of LKM, and 15 who did not, and observed changes in brain imaging during meditation or rest. The study found that when the participants were meditating, the experienced meditators were more sensitive to negative sounds (such as women’s pain) than novice meditators; brain imaging showed activity in the insula, and the study found that this activity was consistent with self-reported increases in altruism. In an in-depth analysis of some participants, the researchers found that activity in the anterior cingulate cortex was also associated with meditation training, particularly in those who had been practicing LKM for a long time. The insula and anterior cingulate cortex, important parts of the central nervous system that detect psychological effects, can reflect the physiological responses of positive emotions (compassion and empathy) and cognitive functions (attention and decision) [60].

LKM did not significantly increase participants’ mindfulness. Mindfulness is active concentration and requires clear internal and external awareness. The related mechanism is one in which the intensity of an individual’s attention control far exceeds that of the event, and during LKM they become completely immersed in the process of communicating positive emotions to themselves and others. In this state of immersion, external consciousness is greatly reduced and a person’s perception of his or her inner self or environment is affected [61]. Previous research has shown that immersion is negatively correlated to mindfulness, which may explain why LKM does not significantly improve mindfulness. Flow is a state of mind in which one is engaged in a certain action. It is a feeling in which one’s mental power is completely invested in a certain activity. The flow experience is accompanied by a high level of excitement and fulfillment. From the perspective of the object, the object of LKM is crude and open. When practicing LKM, the object of meditation is constantly changing, so LKM does not significantly increase the mindfulness of the participants [62].

There was a significant difference in doctor–patient communication ability before and after the test. The first reason for the improvement in doctor–patient communication ability is the improvement of acceptance ability. Acceptance is a positive and defenseless attitude to embrace various experiences; that is, to make room for painful feelings, impulses, and emotions; not to resist, control, or escape them, but to observe them as objects [63]. Acceptance can help physicians to remain open to other perspectives in their interactions with patients, without having to hastily evaluate and categorize incoming information. By focusing in a non-judgmental way, doctors are better able to retain information so they can see its true meaning, rather than being constrained by its response. Thus, they are able to give patients a chance to fully communicate their message and insulate their attention from automatic responses and trivial explanations [64]. Prior to the LKM training, some doctors did not pay attention to the patient’s appeals in the communication process, and interrupted the patient’s narration, which hindered the understanding of some patient conditions and led to the loss of important information. In addition, doctors often adopt one-way communication rather than two-way communication; that is, they do not pay attention to the patient’s response. As a result, the medical staff may not know if the patient fully understands the information conveyed by the communication. These conditions improved after LKM training. The second reason for the improvement in doctors’ ability to communicate is the improvement in their ability to regulate emotions. LKM enables doctors to better deal with negative emotional states and stressful events [65]. As normal social people, medical staff are also subject to emotional anxiety and depression due to work pressure, family conflicts, interpersonal tension, and for other reasons [66]. Emotions play a decisive role in doctor–patient communication. Some physicians, troubled by negative emotions, communicate with patients in a cold, arrogant, and abrupt manner, make treatment plans and medication without explanation, are impatient with patients, and ignore patients’ questions, thus making patients feel unequal. When communicating with patients, they talk in a condescending manner, or use negative, accusatory, and dull words to communicate with patients. As a result, patients experience psychological distress and dissatisfaction, which negatively affects the communication. When medical staff communicate with patients with these negative emotions, the patient’s emotions are directly affected and the effectiveness of doctor–patient communication is negatively affected [7]. LKM enables doctors to better deal with negative emotional states and stressful events. In terms of communication, better emotional regulation should be reflected in the increased ability to remain calm in stressful situations, rather than being overwhelmed by emotions [22].

### 4.2. Research Limitations and Future Studies

This study has the following limitations: (1) The samples were obtained from one hospital, thus, the sample size was relatively small due to the limitations of the number of hospital staff, research funds, and the nature of doctors’ work. (2) Due to the particularity and nature of the doctors’ working environment, the results of this study may not be suitable for extension to other populations, resulting in a low general value of the research results. (3) The analysis of mindfulness, empathy, and doctor–patient communication was undertaken at an overall scale and did not analyze its sub-dimensions. (4) Due to the nature of doctors’ occupations, doctors are busy at work, so it is impossible to put them in a completely closed experimental environment for an 8-week intervention. In addition to LKM intervention during these 8 weeks, subjects also have to continue to participate in daily work and life. Any interference in the intervention experienced by the subjects was beyond the control of this study. (5) The three scales used in this study are all self-reported scales, which are subject to a degree of egocentric error. Thus, the current results should be considered as perceived mindfulness, perceived empathy, and perceived communication skills. (6) Only one model of loving-kindness meditation was used in this study, and a different model might produce different results.

This study explored the effects of LKM on doctors’ mindfulness, empathy, and communication skills, providing a reference for medical educators on how to improve doctors’ communication skills. Clinical instructors can add the relevant content of LKM training to the internship teaching process, such as 10 min of LKM after the end of each shift to cultivate interns’ communication skills. Alternatively, doctors can be guided to re-evaluate their positive and negative emotions to improve their ability to regulate these emotions, with the aim of improving their ability to communicate with patients. In the future, longitudinal studies can be used to explore more relationships between doctor–patient communication variables. Furthermore, we will collect assessments from the perspective of patients or other evaluation subjects to further enhance the objectivity of future research. The clinical communication ability examined in this study has not been previously discussed. Future studies can divide the clinical communication ability into process studies and explorations of the influences of different processes, to provide more effective ideas for improving doctors’ clinical communication ability.

## 5. Conclusions

The current study provides a more detailed and nuanced understanding of the effects of LKM on mindfulness, empathy, and communication skills. The results showed that LKM significantly improved doctors’ empathy and communication skills, but did not significantly promote their mindfulness. This result further elucidates the psychological effects of LKM and raises the possibility of clinical application. The mechanism of LKM’s influence on mindfulness, empathy, communication skills, and other psychological constructs needs to be further clarified.

## Figures and Tables

**Table 1:** Demographic characteristics of participants.

	Total	LKM Group	Control Group
Age (SD)	38.57 (7.41)	37.77 (7.36)	39.36(7.45)
Male (%)	49 (46.2%)	24 (45.3%)	25 (47.2%)
Female (%)	57 (53.8%)	29 (54.7%)	28 (52.8%)

No demographic characteristic was significantly different between the two groups.

**Table 2:** Means and standard deviations for each measure of each group, pre- and post-assessment.

Group	Measures	Mean (SD)		
		Pre	Post	Post-Pre
LKM	MAAS	3.309 (0.675)	3.471 (0.805)	0.162 (0.866)
	JSE	4.826 (0.719)	5.475 (0.859)	0.649 *** (1.237)
	LCSAS	3.283 (0.387)	3.445 (0.415)	1.162 * (0.494)
Control	MAAS	3.214 (0.686)	3.164 (0.719)	−0.050 (0.480)
	JSE	4.916 (0.702)	4.939 (0.776)	0.023 (0.460)
	LCSAS	3.120 (0.441)	3.201 (0.332)	0.081 (1.306)

MAAS, Mindfulness Attention Awareness Scale; JSE, the Jefferson Empathy Scale; LCSAS, Liverpool Communication Skills Assessment Scale. * *p* < 0.05; *** *p* < 0.001.

## Data Availability

Not applicable. The data are not publicly available due to privacy restrictions. Please contact the first author.
